# Copy number variants suggest different molecular pathways for the pathogenesis of bladder exstrophy

**DOI:** 10.1002/ajmg.a.63031

**Published:** 2022-11-08

**Authors:** Agneta Nordenskjöld, Samara Arkani, Maria Pettersson, Johanna Winberg, Jia Cao, Magdalena Fossum, Magnus Anderberg, Gillian Barker, Gundela Holmdahl, Johanna Lundin

**Affiliations:** ^1^ Department of Women's and Children's Health, and Center for Molecular Medicine Karolinska Institutet Stockholm Sweden; ^2^ Pediatric Surgery Astrid Lindgren Children Hospital, Karolinska University Hospital Stockholm Sweden; ^3^ Department of Urology Danderyds Hospital Danderyd Sweden; ^4^ Department of Clinical Genetics Karolinska University Hospital Stockholm Sweden; ^5^ Department of Molecular Medicine and Surgery Karolinska Institutet Stockholm Sweden; ^6^ Department of Pediatric Surgery Copenhagen University, Righospitalet København Denmark; ^7^ Department of Pediatric Surgery Skåne University Hospital Lund Sweden; ^8^ Department of Clinical Sciences Lund University Lund Sweden; ^9^ Department of Pediatric Surgery Uppsala Academic Hospital Uppsala Sweden; ^10^ Sahlgrenska Academy Women's and Children's Health Gothenburg Sweden; ^11^ Department of Pediatric Surgery Queen Silvia's Children's Hospital Gothenburg Sweden

**Keywords:** bladder exstrophy, chromosome, CMA, genetic

## Abstract

Bladder exstrophy is a rare congenital malformation leaving the urinary bladder open in the midline of the abdomen at birth. There is a clear genetic background with chromosome aberrations, but so far, no consistent findings apart from 22q11‐duplications detected in about 2%–3% of all patients. Some genes are implicated like the *LZTR1*, *ISL1*, *CELSR3*, and the *WNT3* genes, but most are not explained molecularly. We have performed chromosomal microarray analysis on a cohort of 140 persons born with bladder exstrophy to look for submicroscopic chromosomal deletions and duplications. Pathogenic or possibly pathogenic microdeletions or duplications were found in 16 patients (11.4%) and further 9 with unknown significance. Two findings were in regions linked to known syndromes, two findings involved the same gene (*MCC*), and all other findings were unique. A closer analysis suggests a few gene networks that are involved in the pathogenesis of bladder exstrophy; the WNT‐signaling pathway, the chromosome 22q11 region, the RIT2 and POU families, and involvement of the Golgi apparatus. Bladder exstrophy is a rare malformation and is reported to be associated with several chromosome aberrations. Our data suggest involvement of some specific molecular pathways.

## 1 INTRODUCTION

Bladder exstrophy is a congenital malformation and part of a clinical spectrum of the bladder exstrophy‐epispadias complex (BEEC, Online Mendelian Inheritance in Man [OMIM] 600057). The phenotypic severity varies from *isolated epispadias*, to the most severe and rare form, *cloaca exstrophy*, with the most common variant *classic bladder exstrophy* (CBE) of medium severity. The incidence of CBE in Sweden is 1 in 30,000 live births (Reinfeldt Engberg et al., 2016). Most cases are sporadic, however, an increased risk in siblings has been noted. Shapiro et al. (1984) reported a recurrence risk for a sibling of 1/70, equivalent to a 400 times increased risk compared with the general population. About 3% of all cases are classified as familial with at least two affected persons in the same family (Reutter et al., 2003). In addition, the pairwise concordance rates in monozygotic as compared with dizygotic BEEC twin pairs are 45% and 6%, respectively (Reutter et al., 2007). Finally, several chromosomal aberrations have been published in association with CBE (Ludwig et al., 2005; von Lowtzow et al., 2016). These combined data strongly suggest an underlying genetic component involving several different genes.

The only recurrent genetic finding so far is the 22q11‐duplication encompassing ~3 Mbp and including 50 genes (Draaken et al., 2010, 2014; Lundin et al., 2010, 2019; Pierquin & Uwineza, 2012). The 22q11 duplication syndrome was initially described in 2008 and was mainly associated with cognitive deficits and dysmorphic facial features. The syndrome has a large clinical variability and reduced penetrance, since healthy and near‐healthy carriers are common (Courtens et al., 2008). Data from different bladder exstrophy cohorts have shown 22q11 duplications in 2.5% of all patients, thus giving an OR of around 30 for being born with bladder exstrophy in carriers. The exact molecular mechanism for how 22q11duplication increases the risk is still unclear. The candidate region was diminished after new findings of smaller regions of overlap and together with expression pattern the main candidate genes are the *CRKL*, *THAP7*, and *LZTR1* genes (Draaken et al., 2014). In addition, our group detected one novel mutation in the *LZTR1* gene located in this region in a patient with CBE and could show that the mutation has functional effect in the Golgi apparatus (Lundin et al., 2019).

Some specific genes have been reported to be associated with BEEC. This includes the *ISL1*‐gene on chromosome 5q11.1, which was identified in a GWAS and with a few mutational findings (Arkani et al., 2018; Draaken et al., 2015). Through whole exome sequencing in CBE a de novo mutation was detected in the *WNT3*‐gene, which also caused defect cloaca development in zebrafish (Baranowska Körberg et al., 2015). Further, three missense mutations have been identified in the *SLC20A1*‐gene on chromosome 2q13, detected in cloacal exstrophy and CBE (Reutter et al., 2016; Rieke et al., 2020). Also, compound heterozygous mutations in the *CELSR3*‐gene, on chromosome 3p21.31, were detected in a child with cloacal exstrophy and other malformations (Reutter et al., 2016).

These findings prompted us to perform a genome‐wide search for deletions and duplications using chromosomal microarray analysis (CMA). The aim of this study was to further evaluate copy number variants (CNVs), in a cohort of 140 well‐characterized Swedish BEEC patients to identify additional genetic events associated with BEEC.

## 2 MATERIAL AND METHODS

### 2.1 Editorial policies and ethical considerations

The study was approved by the Swedish Ethics Review Authority and conforms to the Declaration of Helsinki standards. All patients or parents gave their informed consent prior to inclusion in the study.

### 2.2 Patients

All BEEC patients, adults and children, were recruited from the Pediatric Surgery Departments in Stockholm, Göteborg, Uppsala, and Lund, Sweden. Six patients with previously published chromosome aberrations were excluded from the study (Lundin et al., 2010, 2019; Soderhall et al., 2014). Blood or excess bladder or skin tissue obtained during surgery was collected for DNA isolation and molecular genetic analysis. When possible, parental DNA was also collected.

### 2.3 Chromosomal microarray

A 4 × 180 K custom oligonucleotide microarray with whole‐genome coverage and a median probe spacing of ~18 kb was used (AMADID:031035, Oxford Gene Technology, Begbroke, Oxfordshire, UK). This array design is used as a routine diagnostic tool at the Department of Clinical Genetics, Karolinska University Hospital, Stockholm, Sweden.

The control DNA used for the microarray experiment consisted of a mix of sex‐matched DNA from several healthy individuals pooled together (Promega, Madison, Wisconsin). Sample labelling (CGH labelling kit for oligo arrays, Enzo Life Sciences, Farmingdale, New York), hybridization, and slide washing (Oligo aCGH/ChIP‐on‐Chip Wash Buffer Kit, Agilent Technologies, Wilmington, Delaware) were performed according to the manufacturers' recommendations. Slides were scanned using the Agilent Microarray Scanner (G2505C, Agilent Technologies, USA) with 3 μm resolution. Raw data were normalized using Feature Extraction Software v10.7.3.1 (Agilent Technologies, Santa Clara, California), and log_2_ ratios were calculated by dividing the normalized intensity in the sample by the mean intensity across the reference sample. The log_2_ ratios were plotted and segmented by circular binary segmentation in the CytoSure Interpret software v4.10 (Oxford Gene Technology, Oxfordshire, UK). Oligonucleotide probe positions were annotated according to the human genome assembly hg19 (Genome Reference Consortium Human Build 37). For the 4 × 180 K microarray, three consecutive aberrant probes with a log_2_ ratio cutoff of − 0.65 for deletions and 0.35 for duplications were called, giving a practical lower resolution of about 50 kb. The clinical relevance of all CNVs was classified into five categories: benign, likely benign, variant of uncertain significance, likely pathogenic, and pathogenic, according to the American College of Medical Genetics and Genomics guidelines (Kearney et al., 2011). Classification was based upon the size of aberration, gene content, inheritance, and available information in medical literature and different databases: The Database of Genomic Variants (DGV, 2021), The Database of Chromosomal Imbalance and Phenotype in Humans using Ensembl Resources (DECIPHER, 2021), The Online Mendelian Inheritance in Man (OMIM, 2021), and an in‐house database with variants from >10,000 analyzed clinical patients referred to the Department of Clinical Genetics for CMA. Further information on genes that were involved in deletions or duplications was collected from OMIM, GeneCards—The Human Gene Database (2021), and MalaCards (www.genecards.com; Stelzer et al., 2016).

### 2.4 Fetal data for comparison

RNA expression data obtained from 17 samples of embryonic and fetal bladders (fetal Weeks 5–10) were used for evaluating expression of individual genes in deleted or duplicated CNVs. Data are available publicly at https://www.ebi.ac.uk/arrayexpress/experiments/E‐MTAB‐6592/protocols/.

## 3 RESULTS

### 3.1 Clinical description

We analyzed DNA from 140 individuals with BEEC. Of these, 114 were born with CBE, 14 with epispadias and 12 with cloacal exstrophy and none was a familial finding. Using CMA, we identified 16 microdeletions or duplications (11.4%) that were classified as findings with possible associations to the pathophysiology of BEEC (Table 1). Two were rearrangements in regions previously associated with known syndromes, and 14 were evaluated as likely pathogenic or possibly pathogenic after a standard clinical evaluation. Of these 16, 12 had CBE, one was born with epispadias, two had cloacal exstrophy and in one male the bladder was extremely small, bordering on bladder agenesis. One patient also had cleft lip and palate and one had duodenal atresia. Nine additional samples exhibited CNVs that in a clinical setting would be regarded as of unknown significance (Table 2). Altogether, two patients had two CMA findings (Tables 1 and 2).

**TABLE 1:** Data presenting pathogenic or possibly pathogenic findings in DNA from persons with BEEC

Locus	Position (hg38)	Size	Dup/Del	Phenotype	Sex	Inheritance	Ref Seq genes	Fetal expr	Other db^a^
Two findings in syndromic regions									
16p11.2	chr16:29645396–30168276	523 kb	Del	CBE, ASD	M	NA	**QPRT**, SPN, SEZ6L2, PAGR1, MVP, ZG16, **KIF22**, FAM57B, HIRIP3, TAOK2, GDPD3, **MAZ**, PRRT2, CDIPT, KCTD13, ASPHD, THEM219, **ALDOA**, DOC2A, INO80E, C16orf92, HIRIP3, TBX6, **PPP4C**, YPEL3, **MAPK3**	High expression in bold	Known syndrome
Xq28	chrX:154822249–155197455	375 kb	Dup	CBE and duodenal stenosis	F	NA	SMIM9, F8, **FUNDC2**, CMC4, MTCP1, **BRCC3**, **VBP1**	Yes, all	Known syndrome
Possibly pathogenic findings									
1p36.11	chr1:26002697–26255559	253 kb	Del	CBE	M	Mat	EXTL1, SLC0A2, TRIM63, PDIK1L, FAM110D, ZNF593, CNKSR1, CATSPER4, CEP85	Yes, all	Novel
3q26.1	chr3:160985377–164524799	3.54 Mb	Dup	Epispadias	M		**PPM1L, B3GALNT1, NMD3**, SPTSSB	Yes	A few similar reported
5q22.2	chr5:113180369–113318123	138 kb	Del	CBE	M		**MCC**	Yes	Only larger del reported
5q22.2	chr5:113001507–113151488	150 kb	Dup	Cloacal exstropy and cleft palate	F	Pat	**MCC**	Yes	Only larger dup reported
6q23.2	chr6:133892118–133892292	175 kb	Dup	CBE	F		**TCF21**	Yes	Novel
8q22.2	chr8:98278761–98644873	366 kb	Del	CBE	F	Pat	NIPAL2, KCNS2, **STK3**	Yes	One smaller reported
8q22.2	chr8:99529972–99624795	95 kb	Del	CBE	M	Mat	**VPS13B**	Yes	Similar reported^b^
9p13.2	chr9:36363506–36944808	581 kb	Dup	CBE	F	Mat	RNF38, **MELK**, PAX5	Yes	Three similar reported
9p22.2	chr9:18351191–18668331	317 kb	Dup	CBE	F	Pat	ADAMTSL1	Yes	A few reported
10p12.2	chr10:24091999–24375694	284 kb	Dup	Bladder agenesis	M	Pat	KIAA1217	Yes	A few reported
16q24.3	chr16:89752071–89842991	91 kb	Del	CBE	M	Mat	FANCA, SPIRE2	Yes	Reported earlier
18q12.3	chr18:43052727–43463356	411 kb	Del	CBE	M	Pat	RIT2, **SYT4**	Yes	Novel
21q22.12	chr21:34671646–34731540	60 kb	Dup	CBE	F^c^	Mat	CLIC6	Yes	Novel
Xq23	chrX:115139141–115189073	50 kb	Del	CBE	M	Mat	LRCH2	Yes	Novel

*Note*: Genes in bold have a high expression in fetal bladder, gestational weeks 5–10 (Expression Atlas ‐ E‐MTAB‐6592).

Abbreviations: ASD, autism spectrum disorder; BEEC, bladder exstrophy‐epispadias complex; CBE, classic bladder exstrophy; DECIPHER, The Database of Chromosomal Imbalance and Phenotype in Humans using Ensembl Resources; DGV, The Database of Genomic Variants; OMIM, The Online Mendelian Inheritance in Man.

^a^ Searches for similar variants have been performed in DGV, DECIPHER, OMIM and our in‐house database with variants from >10,000 analyzed clinical cases referred for CMA as reported in January 2022.

^b^ Two cases with urogenital malformations among those reported.

^c^ Same individual with two findings, Tables 1 and 2.

**TABLE 2:** Data presenting findings in DNA from persons with BEEC that would be regarded as of unknown significance

Locus	Position (hg38)	Size (kb)	Dup/Del	Phenotype	Sex	Inheritance	Ref Seq genes	Fetal expr	Detected in other db^a^
2q35	chr2:220073652‐220182172	109 kb	Del	Cloacal exstrophy	M		ZFAND2B, ABCB6, ATG9A, ANKZF1, GLB1L, STK16, TUBA4A, DNAJB2, and PTPRN	Yes	Less than 10 similar
3p25.3	chr3:10259315–10285321	25	Del	CBE	M^b^		TATDN2	Yes	Three larger reported
3q21.1	chr3:123180879–123276259	95	Del	CBE	F^c^		SEC22A	Yes	Less than 10 similar
4p16.3	chr4:342319–426951	85	Del	CBE	F		**ZNF141**	Yes	Similar reported
7p22.2	chr7:3406217–3642243	236	Dup	CBE	M	Mat	SDK1	Yes	Several reported
8p23.1	chr8:11809436–11875305	66	Del	CBE	F	Pat	**FDFT1** and **CTSB**	Yes	Around 10 reported
8p22	chr8:15218133–15334651	117	Del	Cloacal exstrophy	M		SGCZ	Yes	Several reported in the region
9q31.1	chr9:99907569–99959918	52	Dup	CBE	F		STX17	Yes	Only two reported
19q13.2	chr19:40819832–41120423	301	Dup	CBE	M^b^	Pat	BCKDHA, THEM91, ERICH4, DMAC2, B9D2, TGFB1, EXOSC5, B3GNT8, TPM3P5, and CEACAM21	Yes, apart from ERICH4	Around five reported

*Note*: Genes in bold have an established expression in fetal bladder gestational week 5–10 (Expression Atlas ‐ E‐MTAB‐6592).

Abbreviations: BEEC, bladder exstrophy‐epispadias complex; DECIPHER, The Database of Chromosomal Imbalance and Phenotype in Humans using Ensembl Resources; DGV, The Database of Genomic Variants; OMIM, The Online Mendelian Inheritance in Man.

^a^ Searches for similar variants have been performed in DGV, DECIPHER, OMIM, and our in‐house database with variants from >10,000 analyzed clinical cases referred for CMA as reported in January 2022.

^b^ Same individual with two findings, Table 2.

^c^ Same individual with two findings, Tables 1 and 2.

### 3.2 CNV findings

Data on microarray findings are listed in Tables 1 and 2, including chromosomal region, size of the deletion/duplication, phenotype, sex, parental status when available, genes within the affected region, and whether the genes within the region are expressed in human fetal urinary bladder. Information on genes was collected from OMIM and GeneCards as described above. The aberrations detected in the study were not recurrent apart from 5q22.2 where two cases had aberrations (one deletion and one duplication) involving different parts of the same gene, the MCC Regulator of Wnt Signaling pathway gene, *MCC*.

### 3.3 Two findings in syndromic regions

**FIGURE 1:**
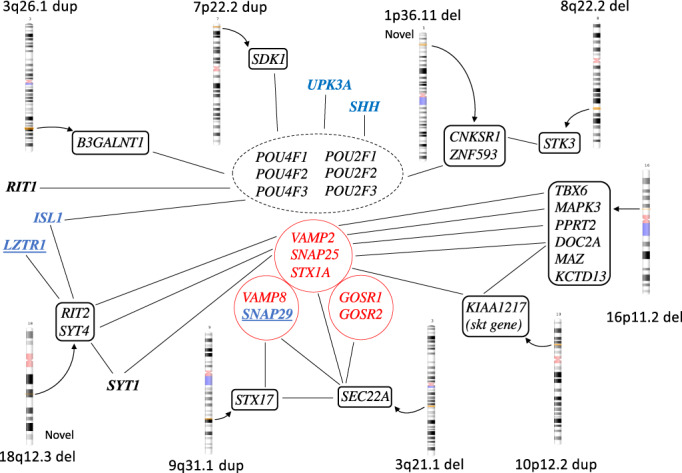
Network data from GeneCards on genes located within nine of the copy number variants (CNVs) presented in this study and highlighting possible connections (black lines) concerning networks with the POU‐gene family and Golgi‐associated genes, respectively. CNVs in black boxes with affected genes in italic within the box. Genes described in MalaCards as associated with bladder exstrophy‐epispadias complex in blue. Candidate genes in the 22q11‐region are underlined in blue. SNARE‐proteins important for the Golgi apparatus function are marked in red.

**FIGURE 2:**
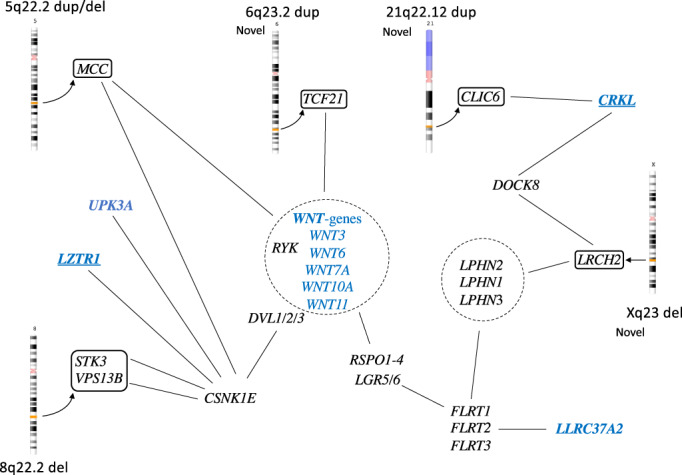
Network data assembled from GeneCards on genes located within six of the copy number variants (CNVs) presented in this study and highlighting possible connections (black lines) concerning the *WNT* gene family and latrophilins. CNVs in black boxes with affected genes in italic within the box. Genes described in MalaCards as associated with bladder exstrophy‐epispadias complex in blue. Candidate genes in the 22q11‐region are underlined in blue.

One DNA sample showed a “16p11.2 deletion” (523 kb), encompassing 24 known genes and covering almost the whole region of the classic 16p11.2 deletion syndrome (Table 1, Figures 1 and 2). This contiguous gene syndrome typically covers 593 kb and is often associated with hypospadias and other forms of urogenital malformations (Nik‐Zainal et al., 2011; Sampson et al., 2010; Tannour‐Louet et al., 2010). Among those with urogenital malformations, the smallest common region of 217 kb contains the MYC‐Associated Zinc Finger Protein, *MAZ*, that is a dosage‐sensitive regulator in urogenital development and crucial for normal bladder development (Haller et al., 2018). Our case with a 523 kb deletion and a neuropsychiatric disorder is the first reported with bladder exstrophy. The mechanism could be associated with *WNT*‐gene expression. The deleted region also contains two additional genes that are potentially interesting. The T‐box transcription factor 6 gene (*TBX6*), is involved in the fetal development in early mesoderm and described as the driver for renal and urinary tract malformations in the 16p11‐deletion syndrome (Verbitsky et al., 2019). The Potassium Channel Tetramerization Domain‐containing protein 13 (*KCTD13*) gene is expressed in fetal bladder and may also be a candidate for urogenital malformations. There are also other genes highly expressed in fetal bladder in this region, marked in bold in table 1. The Double C2‐like domain‐containing protein alpha, *DOC2A*, is involved in fusion between membranes. It is localized mainly in the nucleus and is associated with several Golgi proteins like SNAP25, SNAP29 (in the 22q11 duplication region), and STX1B (Sato et al., 2010, GeneCards).

One female with CBE in combination with duodenal stenosis carried a 375 kb duplication on chromosome Xq28 containing only a few genes: Small integral membrane protein 9 (*SMIM9*), Coagulation factor VIII (*F8*), FUN14 Domain Containing protein 2 (*FUNDC2*), and C‐X9‐C Motif containing 4 (*CMC4*), Mature T‐cell proliferation 1 (*MTCP1*), and the BRCA1/BRCA2‐containing complex, subunit 3 (*BRCC3*). In most patients, the Xq28 duplication syndrome covers a larger region and includes the gene *MECP2*, that is regarded as causative of the intellectual disability and cognitive disturbances. The syndrome has been described as associated with bladder dysfunction (Clayton‐Smith et al., 2009).

### 3.4 CNV‐findings regarded as possibly pathogenic

One male with a CBE carried a maternally inherited 1p36.11 deletion (253 kb), involving nine genes. This deletion is not described in any public databases. Three of the genes are active in skeletal or muscle tissue that could be involved in the pathogenesis in BEEC, namely, the Exostosin‐like glycosyltransferase 1 (*EXTL1*) the Tripartite Motif‐Containing protein 63 (*TRIM63*), and the Centrosomal protein, 85‐KD, *CEP85* genes. The gene products of *EXTL1* and *CEP85* are mostly localized in the Golgi apparatus. The Zinc‐Finger Protein 593 (*ZNF593*) gene is a negative regulator of *OCT2* (the *POU2F2* gene; Terunuma et al., 1997).

One larger 3q26.1‐duplication (3.54 Mb), containing four genes, was identified in a male with epispadias. A few similar duplications are reported in databases. All the duplicated genes are expressed in fetal urinary bladder, supporting the hypothesis that one or more of the genes contributed to the malformation. The NMD3 ribosome export adaptor (*NMD3*) is located in the nucleus. The Serine Palmitoyltransferase, small subunit B (*SPTSSB*) gene is associated with Golgi‐proteins. The Protein Phosphatase, magnesium or manganese requiring protein (*PPM1L*), is expressed in muscle. It is involved in initiating an apoptosis cascade and associated with Golgi function. The beta‐1,3‐*N*‐acetylgalactosaminyltransferase 1 gene (*B3GALNT1*) is highly expressed in the Golgi apparatus, and is active in embryonal ectoderm, in urinary bladder and is associated with the *POU2F3* gene.

On chromosome 5q22.2, aberrations were detected in two patients, one with CBE and one with cloacal exstrophy. Both aberrations, one paternally inherited 150 kb duplication and one 138 kb deletion of unknown inheritance, were in the same gene but not covering overlapping regions. The gene MCC Regulator of Wnt Signaling pathway, *MCC*, is a tumor suppressor gene and somatic mutations have been detected in colorectal cancer. The gene is expressed in urinary bladder and this region is also described in bladder cancer progression (Majewski et al., 2008; von Knobloch et al., 2000).

One interesting duplication finding on chromosome 6 (6q23.2) contains the 3′ end of the gene Transcription Factor 21 (*TCF21*), that is active in the embryological mesoderm surrounding the genitourinary system and a prognostic marker in bladder cancer (Lotfi et al., 2021). This duplication has not been described previously. The gene is associated with both canonical and non‐canonical WNT‐pathways as well as with the *ALDOA* gene, in the 16p11.2 deletion, described above.

Two non‐overlapping 8q22.2 deletions were found in a male and a female with CBE. One was a unique 366 kb deletion containing three genes. First, the NIPA Like Domain Containing 2, *NIPAL2*, which codes for a membrane protein, earlier named *SLC57A4*, that has been associated with ichtyosis. Second, the Potassium Channel, Voltage‐Gated, Delayed‐Rectifier7, Subfamily S, Member 2, *KCNS2*, which codes for a membrane protein only expressed in brain tissue. Last, the Serine/Threonine Protein Kinase 3 (*STK3*), a growth suppressor that is expressed during human fetal development, also in urinary bladder, and especially important for vasculogenesis. *STK3*, together with its homolog *STK4*, is part of the Hippo pathway that is important during development for growth and apoptosis. The *STK3/4* genes are also associated with the *CNKSR1* gene in the chromosome 1p36.11‐deletion. All three deleted genes are expressed in fetal bladder, although *KCNS2* to a small degree. This deletion was also found in the father.

The second 8q22.2 deletion was 95 kb, inherited from the mother and involved only one single gene, the Vacuolar Protein Sorting 13 homolog B, *VPS13B*. It is expressed in most tissues including fetal urinary bladder, and mutations cause the autosomal recessive Cohen syndrome with intellectual disability, microcephaly, and epilepsy, as well as other symptoms. VPS13B is a transmembrane protein important in vesicle‐mediated transport (Rodrigues et al., 2018), maybe post‐Golgi. In vitro knock‐down has shown alterations in the Golgi, lysosome and endosome morphology (Duplomb et al., 2014). Gains of the 8q22 region have been described in bladder cancer (Richter et al., 1999). This patient also carried a previously reported *ISL1*‐mutation (Arkani et al., 2018). Similar deletions have been reported in‐house in two patients with gastrointestinal and kidney malformations.

Three genes reside within the 9p13.2 duplication (581 kb) that was found in a female with CBE and her mother. The Ring Finger Protein 38, *RNF38*, is expressed in different tissues and active in development and oncogenesis as well as regulation of p53 (Sheren & Kassenbrock, 2013). The Maternal Embryonic Leucine Zipper Kinase, *MELK*, codes for a protein kinase that is important in early embryonic development for example in skeletal muscle and during oncogenesis (Jiang & Zhang, 2013). Both these genes are highly expressed in fetal bladder. The Paired Box Protein 5, *PAX5*, is crucial for B‐cell differentiation. Similar aberrations have been reported in a few cases in public databases.

The 9p22.2p22.1 duplication (317 kb), found in a female with CBE only encompasses the gene Adamts‐like Protein 1, *ADAMTSL1*, which is initially expressed at a low level in fetal bladder, increasing over time during bladder development. The glycoprotein is found extracellularly and in the endoplasmatic reticulum. The gene is expressed in skeletal muscle and may be involved in a midline orientation network (Seetharaman et al., 2011). Interestingly, CBE has also been described in a case of Opitz syndrome, a midline disorder (Jacobson et al., 1998). A few cases have been reported in public databases. The duplication in our patient was found paternally inherited.

The gene *KIAA1217* (Sickle tail, mouse, homolog of, *SKT*) is duplicated in the chromosomal region 10p12.2p12 (284 kb). Such a duplication was not found in the in‐house database, but a few in public databases. The duplication was paternally inherited. The gene is expressed in many tissues, especially in intervertebral discs. *KIAA1217* corresponds to the sickle tail (*skt*) gene in mice and is expressed also in the cloacal plate during fetal development (Semba et al., 2006; Suda et al., 2011). This gene is of special interest due to association and network with the paralogue gene, SRC Kinase Signaling Inhibitor 1, *SRCIN1*, and *SNAP25*, and further to the *SYT*‐gene family as well as to the *DOC2A* gene on chromosome 16p11.2‐region (Figure 1; www.genecarsd.org). The SRCIN1 protein is part of the cytoskeleton, and the function leads to impaired cell spreading and migration, and it is also involved in exocytosis. It is not normally expressed in fetal bladder. The *SNAP25* gene is expressed during the fetal bladder development.

The 16q24.3 deletion (91 kb), contains the gene for Fanconi anemia, complementation group A (*FANCA*), and the gene Spire‐type Actin Nucleation Factor 2, *SPIRE2*. Fanconi anemia is a clinically heterogenous autosomal recessive disorder associated with congenital malformations in about 70% of the patients. However, no association has previously been described with bladder exstrophy. Our patient was a male with CBE. The deletion was maternally inherited. The *SPIRE2* gene is not very well characterized but is involved in intracellular vesicle transport along actin fibers. Similar chromosome aberrations have been reported in other databases.

One male born with CBE carried a paternally inherited deletion on chromosome 18q12.3 (411 kb), involving only the two genes Ras Like Without CAAX 2, *RIT2*, and Synaptotagmin 4, *SYT4*. The *RIT2* gene is a small GTP‐binding protein in the Ras family and is expressed in fetal bladder during the fetal Weeks 5–10 at low levels. Interestingly, the *RIT2* gene is also associated with two earlier identified BEEC‐genes, the Leucine Zipper Like Transcription Regulator 1, *LZTR1* gene on chromosome 22q11.2 and the ISL Lim Homeobox 1, *ISL1*, gene (GeneCards, Zhang et al., 2013). The *ISL1*‐gene can modify POU4F1 binding sites in the *RIT2* promotor region and modulate the activity of the POU4 factors. The POU4F1 is a highly conserved transcription factor expressed in the early embryo.

The *SYT4* gene is expressed in neural tissue and in fetal bladder. There is an association with the *SYT 1* and *4* with the *PPRT2* and *DOC2A*‐ genes, located in the 16p11.2 deleted region, through the Synaptosomal‐Associated protein 25, *SNAP25*, gene. The function is membrane fusion and exocytosis. The *SNAP25* gene is one of three SNARE proteins in the Golgi apparatus, the others being syntaxin 1A, *STX1A*, and synaptobrevin or vesicle‐associated membrane protein 2 (*VAMP2*), all three connected to the *SYT1* gene. Recently an association to the *SYT1* gene, on chromosome 2q21.2, was identified in a Genome Wide Association Study (GWAS) of BEEC (Reutter et al., submitted).

One 50 kb duplication that only covered one single gene, the Chloride Intracellular Channel 6, *CLIC6*, was detected on chromosome 21q22.12. This gene is expressed in fetal bladder. The protein is located in the plasma membrane and extracellularly. There is an association with the genes *YES1* and *FYN* and thereby also with three POU‐genes (*POU2F3*, *POU4F1*, and *POU5F1*), and the *SRCIN1* gene, mentioned due to an association with the *KIAA1217* gene on chromosome 10.

The Xq23 deletion (50 kb) has not been reported earlier and covers only one single gene, Leucine Rich Repeats and Calponin Homology Domain Containing 2 (*LRCH2*). The deletion was detected in a male with CBE and is maternally inherited, which may be consistent with an X‐linked recessive trait. The gene is highly conserved from *Drosophila* and regulates cytoskeleton scaffolding (Foussard et al., 2010). The protein interacts with both a family of Latrophilins (LPHN1‐3) LPHN1‐2‐3 and the Dedicator of cytokinesis protein 8, *DOCK8*, and the Actin‐related protein 2/3 complex subunit 3, *ARPC2*, genes. Both the *LPHN2* gene (1p31.1) and the *ARPC2* gene are highly expressed in fetal bladder. The *LPHN2* gene or Adhesion G protein‐coupled receptor L2, *ADGRL2* gene is located on chromosome 1p31.1 and its function involves cell adhesion and exocytosis. The network includes Fibronectin Leucine Rich Transmembrane Protein, *FLRT1*, *2*, and *3* and the Leucine Rich repeat Containing G Protein‐Coupled Receptor 5 (*LGR5*), gene that is a link also to WNT‐signaling via R‐spondins, *RSPOND1* and 2.

### 3.5 Additional findings of unknown significance

Some further findings were evaluated as likely benign given current knowledge based on findings in other disorders or malformations in a clinical setting. They could however still be interesting as parts of different networks or in the pathogenesis of BEEC.

In the deleted region on chromosome 2q35, the Serine/ Threonine protein Kinase 16 gene, *STK16*, is located. It is expressed in the Golgi apparatus and involves vesicle trafficking. The deleted region on chromosome 3 (3q21.1) contains the SEC22 Homolog A, Vesicle Trafficking Protein gene, *SEC22A*. This gene is also expressed in the Golgi apparatus and the endoplasmatic reticulum, functions in vesicle trafficking and is associated both with SNAREs complex and other Golgi‐located proteins (String network; Szklarczyk et al., 2021). It is further expressed in fetal bladder.

One girl with isolated CBE carried a small 4p16.3 deletion inherited from the father and encompassing only the *ZNF141*‐gene. This region is part of the deleted region in Wolf‐Hirschhorn syndrome (WHS; Battaglia et al., 2015). The smallest overlapping region is published as 2.6 Mb, involving almost 50 genes including the *ZNF141‐gene* (Yang et al., 2016). About one third of WHS patients also have urogenital malformations (Battaglia et al., 2008). An association between BEEC and WHS has been described in one case with epispadias and in one among 34 patients with CBE (Nicholls & Duffy, 1998; Schwanitz & Grosse, 1973). The importance of this continuous gene syndrome in bladder disorders was further supported by the finding of somatic *FGFR3* mutations in one 8 years old WHS patient with hematuria and a rare form of myofibroblastic bladder tumor (Marte et al., 2013). The region is also important in bladder cancer progression (Bell et al., 1996; Bell et al., 1997; Cappellen et al., 1999; di Martino et al., 2013; Elder et al., 1994; Rothman et al., 2010; Sibley et al., 2000). Mutations in the *ZNF141* gene have been described in one family with polydactyly and it is regarded as a tumor suppressor gene (Kalsoom et al., 2013). Comparable small deletions of *ZNF141* were detected in 0.02% of 20,000 healthy controls.

The Sidekick cell Adhesion Molecule 1 (*SDK1*), gene in the duplicated region on chromosome 7p22.2 is expressed in fetal bladder and is associated both with SNAREs complex and carry binding sites for the *POU6F1* gene. The main function is reported to be tricellular adhesion (Letizia et al., 2019). Several deletions are reported in public databases Another potentially interesting finding is the duplication on 9q31.1 involving the Syntaxin 17, *STX17*, gene, active in membrane fusion and associated with SNAREs proteins and Golgi proteins such as Golgi SNAP receptor complex member 1, GOSR1. Similar array findings have been reported twice in public databases.

## 4 DISCUSSION

We have previously identified altogether five typical 22q11‐duplications associated with BEEC (Lundin et al., 2010, 2019). The prevalence in our material is 3.4% in reported BEEC patients as compared with 0.08% in controls (Lundin et al., 2010). We have also reported one family with an X‐chromosome rearrangement (Soderhall et al., 2014). Now we have extended the analysis with CMA, using DNA from a further 140 BEEC patients, to try and identify other candidate regions for BEEC. Submicroscopic duplications/deletions possibly associated with BEEC were identified in 16 individuals (11.4%). Earlier studies using CMA have detected novel possibly pathogenic microduplications/ deletions in 9% (10 of 110) and 8% (13 of 169), respectively (Draaken et al., 2013; von Lowtzow et al., 2016). Recently, data on exome sequencing in trios with BEEC were published reporting three gene variants in the *WNT3* gene and in the 22q11‐duplicated region (*LZTR1* and *CRKL* genes), together with new candidate genes and CNVs (Pitsava et al., 2021).

What is most striking is that studies so far have very few overlapping findings and our data on CNVs show almost no overlap within or compared with other studies. That mutations have been reported in several different genes supports the notion that BEEC has a complex genetic background with the involvement of many different genes as well as possibly environmental factors involved (Reutter et al., 2016). This fits well with the epidemiological data and increased recurrence risks, that do not fit Mendelian inheritance. It is therefore possible that several different gene variants in the same patient may be risk factors. In that case, it would be expected that genetic aberrations detected in affected individuals would be inherited, since one mutation alone may not suffice to cause the disease. Instead, the sum of genetic events inherited from the two parents, or de novo events, sometimes combined with an environmental factor could cause the malformation. It is further likely that genetic variants are instead linked via different molecular pathways that can be disrupted during development, causing BEEC. As outlined in the result section, genes that are identified in this study are associated in networks and pathways. All genes that we highlight here are in addition expressed in human fetal urinary bladder tissue, indicating a role in bladder development and consistent with a role in BEEC pathogenesis.

### 4.1 Novel findings

Five findings were novel and unique in our study, 1p36.11, 6q23.2, 18q12.3, 21q22.12, and Xq23, and therefore may be of particular interest. The 1p36.11 deletion involves genes that are active in both muscle and skeleton including the *ZNF593* gene which negatively regulates POU2F2, part of the Pit‐Onc‐Unc (POU)‐gene family (Hayes et al., 2008; Terunuma et al., 1997). The POU protein family of transcription factors originates from Pit‐1, Oct‐1, and Oct‐2, that are highly conserved from Drosophila, and consists of 6 classes and 15 genes. Their fundamental function is to orchestrate embryonic development and to direct cellular fate decisions (Malik et al., 2018). Thirteen of the *POU*‐genes are expressed in fetal bladder, apart from the POU1F1 and POU4F3, with POU2F1 being the most highly expressed early in development in ectoderm and epidermis. Interestingly, genes in two of the CNVs, 3q26.1, 18q12.3, are associated with the POU networks. Finally, mutations in three different BEEC genes (the *ISL1*, *UPK3A*, and *LZTR1*) have been reported earlier and they are also associated with this network. Such data suggest that especially the POU2F and POU4F networks may be important during fetal bladder development. The *TCF21* gene (6q23.2) is active in the embryonal mesoderm surrounding the genitourinary system and is also involved in bladder cancer (Lotfi et al., 2021). Other novel candidate genes are the *RIT2* and *SYT4* genes and the *CLIC6* as well as the LRHC2 gene (Xq23). The latter links to latrophilins (LPHN1‐3), a family of cell‐adhesion receptors, and WNT signaling pathways. The *LPHN1‐3* gene family is expressed in fetal bladder, with especially high levels of *LPHN2*, and these genes are also linked to the gene *LLRC37A2* that is listed as a candidate gene to BEEC (MalaCards).

### 4.2 Findings partly overlapping with earlier studies; the MCC‐gene, WNT gene involvement, and the 16p11.2 chromosome region

The *MCC* gene was involved in two patients, one duplication and one deletion involving different parts of the gene. This gene has previously been reported associated with BEEC due to the location within a larger paternally inherited duplicated region that also included five other genes (von Lowtzow et al., 2016). We have no in‐house findings although a few have been reported in public databases. The gene is highly expressed in the human fetal bladder and is active in regulation of the WNT signaling pathway, thus highlighting the importance of WNT in urogenital development. Homozygous *WNT3* stop mutations (Q83X) were reported in a large consanginous family with tetra‐amelia in three fetuses (Niemann et al., 2004). In two of these, hypoplasia of the pelvis was detected, and one also had persistence of cloaca in addition to other malformations. We have previously reported a de novo *WNT3* mutation associated with bladder exstrophy which caused cloaca malformation in zebrafish (Baranowska Körberg et al., 2015). In addition, the first GWAS conducted on BEEC showed an association to a region near the *WNT3* and *WNT9b* genes (Reutter et al., 2014). According to GeneCards, other genes possibly involved in these networks are the *CSNK1E*, *RSPO1‐4*, and *LGR5*‐genes.

The 16p11.2 deletion syndrome (523 kb) in our study is similar to the duplicated CNV published by Pitsava et al. (2021) which was 733 kb and partly overlapping. At least in individuals carrying 16p11.2 deletions, urogenital malformations are common.

### 4.3 Associations with genes in the 22q11‐region

In the implicated 22q11‐region, the genes that most likely function as risk factors for BEEC according to the smallest region of overlap are the *CRKL*, *AIFM3*, *LZTR1*, and *THAP7* genes (Draaken et al., 2014). The *LZTR1* gene is mainly localized in the Golgi apparatus and helps stabilize the Golgi complex. We have earlier reported a mutation in the *LZTR1* gene when an inactivating mutation in *LZTR1* gene resulted in defective exocytosis (Lundin et al., 2019). One of the two genes presented here in the 18q12.3 deletion is the *RIT2* gene. This gene is novel as a candidate for BEEC. It is expressed in fetal bladder but is also associated with two earlier recognized BEEC genes, both the *LZTR1* gene in the 22q11 duplication region, together with the paralog RIT1, as well as the *ISL1* gene. The *RIT2* gene is neuron specific in mouse retina cells, and it was shown that its promotor was regulated by POU4 TFs, but in addition, ISL1 could modulate this activity (Zhang et al., 2013). The *RIT2* gene is also associated with the POU‐family genes *POU4F1* and *POU4F2*.

The *LRCH2* gene in the Xq23 deletion functions to stabilize cell cortex during cell division as a cytoskeletal scaffolding protein (Foussard et al., 2010). From an earlier WES, we found one frameshift mutation in this gene in one patient with CBE (Baranowska Körberg et al., 2015, unpublished data). The gene is associated with the genes LPHN1‐2‐3 and in a network with the DOCK8 gene as well as the *CRKL* gene on chromosome 22.

### 4.4 Involvement of the Golgi complex

The Golgi apparatus is a central intracellular membrane‐bound organelle and involved in both trafficking, cell polarity, and signaling in the cell. Proteins produced in the ER must be compartmentalized and transported in vesicles within the Golgi apparatus, to the cytosol, or to the cell membrane for secretion (Ravichandran et al., 2020; Sun et al., 2020; Tang, 2021). This process is handled by many proteins in complexes called Soluble *N*‐ethylmaleimide‐sensitive factor attachment protein receptor, SNAREs, that mainly include the Vesicle Associated Membrane Protein 2 (VAMP2), Synaptosome Associated Protein 25 (SNAP25), and Syntaxin1A (STX1A), but also Syntaxin‐5 (STX5), Golgi SNAP Receptor Complex member 1 and 2 (GOSR1, GOSR2), SEC22 Homolog B, Vesicle Trafficking Protein (SEC22B), and Bet1 Golgi Vesicular Membrane Trafficing Protein (BET1). One other important function for the Golgi apparatus is to direct cell polarity while positioning in relation with the nucleus. This function is for example crucial during migration (Ravichandran et al., 2020). As discussed above, many genes are involved in the Golgi function such as the *LZTR1*, *SYT4*, *SYT1*, *SEC22A*, and *KIAA1217*, as well as genes in the 16p11.2 deletion (*DOC2A*, *PPRT2*).

In addition to the chromosomal findings described in this article we want to emphasize that the task to evaluate pathogenicity of these CNVs is still challenging. CMA is routinely used in clinical genetic diagnostics, which improves the quality of the evaluation of the analysis and findings are evaluated against a growing number of samples. The strength of this study is that it is large, with well‐characterized phenotypes. A limitation is that we did not have parental DNA in all patients. Thus, given disparate findings, further studies are needed to understand the pathogenesis of BEEC.

In conclusion, in this study, we performed CMA on DNA from 140 BEEC patients and found possibly pathogenic CNVs in 16 cases. Previous studies and this study have found inactivating mutations or CNVs involving several different genes expressed in human fetal bladder. The number of genes is consistent with a polygenic mechanism for the malformation. We wanted to highlight genes that seem to be involved in networks critical for early human urinary bladder development. Extensive mutation screening has so far only been reported in a limited number of patients, and one would expect that future extensive sequence analysis of BEEC patients and parents will shed more light on the mechanisms behind BEEC. In clinical praxis, it would be advantageous if CMA was performed on all children born with BEEC, both given that around 10%–15% will have a finding that could have clinical relevance and that this will further increase our knowledge about the pathogenesis.

## AUTHOR CONTRIBUTIONS

*Project management*, *initiation of the study, and funding*: Agneta Nordenskjöld. *Planning*: Agneta Nordenskjöld and Johanna Lundin. *Case recruitment and phenotype characterization*: Agneta Nordenskjöld, Samara Arkani, Magdalena Fossum, Magnus Anderberg, Gillian Barker, and Gundela Holmdahl. *Experimental work*: Johanna Lundin, Jia Cao, Maria Pettersson, and Johanna Winberg. *Data analysis*: Johanna Lundin, Samara Arkani, and Agneta Nordenskjöld. Writing article draft: Agneta Nordenskjöld, Johanna Lundin, and Samara Arkani. Input on article: all authors.

## FUNDING INFORMATION

Swedish Research Council (grant K2012‐64X‐14506‐10‐5 2016–01642, to Agneta Nordenskjöld), Foundation Frimurare Barnhuset Stockholm, Stockholm City Council, Karolinska Institutet, the Swedish Brain Foundation, the Harald and Greta Janssons Foundation, and Erik Rönnbergs Foundation.

## CONFLICT OF INTEREST

The authors declare that there is no conflict of interest that could be perceived as prejudicing the impartiality of the research reported.

## Data Availability

The data that support the findings of this study are available on request from the corresponding author. The data are not publicly available due to privacy or ethical restrictions.
